# Synergy Effect of Nano-Organic Palygorskite on the Properties of Star-Shaped SBS-Modified Asphalt

**DOI:** 10.3390/polym13060863

**Published:** 2021-03-11

**Authors:** Shuai Liu, Yuchao Gao, Jiao Jin, Huiwen Chen, Xinyu Liu, Ruohua Liu, Qingjun Guan, Yinrui Wu, Huaqiang Long, Guoping Qian

**Affiliations:** 1 School of Traffic and Transportation Engineering, Changsha University of Science and Technology, Changsha 410114, China; liushuai1205468383@stu.csust.edu.cn (S.L.); gaoyuchao@stu.csust.edu.cn (Y.G.); chenhuiwen@stu.csust.edu.cn (H.C.); liuxinyu102401@sina.cn (X.L.); wyr@stu.csust.edu.cn (Y.W.); 201902140520@stu.csust.edu.cn (H.L.); guopingqian@sina.com (G.Q.); 2 School of Minerals Processing and Bioengineering, Central South University, Changsha 410083, China; ruohualiu@csu.edu.cn; 3 School of Resource Environment and Safety Engineering, Hunan University of Science and Technology, Xiangtan 411201, China; guanqingjun@hnust.edu.cn

**Keywords:** palygorskite, SBS, modified asphalt, rheological properties

## Abstract

With the rapid development of economic construction, styrene-butadiene-styrene (SBS)-modified asphalt is being more and more widely used in highway engineering, but there are still many deficiencies in the process of its use. In order to further improve its performance for use, nano-organic palygorskite (A-Pal) and star-shaped SBS were compounded to obtain modified asphalt in this study. The high-temperature stability of SBS-modified asphalt was enhanced after incorporation with A-Pal for the high-temperature stability test by a dynamic shear rheometer. The A-Pal should improve the surface free energy and adhesion of SBS-modified asphalt by the water stability test analysis. The aging test shows that A-Pal can reduce the thermal oxygen decomposition of SBS and improve the anti-aging performance and the fatigue resistance of SBS-modified asphalt. A-Pal has a certain improvement effect on the low temperature performance of SBS-modified asphalt as shown by a low temperature crack resistance test. A-Pal-compounded SBS-modified asphalt features good storage stability in normal temperatures with the lowest critical compatibility temperature.

## 1. Introduction

In recent years, nano-materials and nano-technology have been applied more frequently in the field of pavement traffic materials, and nano-modified asphalt has become one of the hot topics of research [1,2,3,4]. At present, nano-layered silicate materials are generally applied to asphalt materials because of the large output and good performance [5,6,7]. The nano-layered silicate with a special crystal structure, which makes asphalt molecules enter the layered structure, can increase the layer spacing, improving the form peel structure, which prevents oxygen from penetrating into the asphalt and delaying its aging. Therefore, the nano-modified asphalt has good anti-rutting and anti-aging properties [8,9,10]. At the same time, organic modified nano materials can also improve the dispersion degree of polymer in asphalt, providing broad development prospects for modified asphalt in the future.

Palygorskite (Pal), also known as attapulgite, is a layered chain water-rich magnesium–aluminum silicate clay mineral. It has a reputation as “the king of earth” for its wide range of applications. The crystal structure of Pal is characterized by the double-layer Si-O tetrahedral sheets that are connected with the single-layer (Mg, Al)-O octahedron sheets and the unit layers are connected by oxygen to form a pore-like crystal structure [11]. The pores are filled with zeolite water and crystal water to form a fibrous single crystal. The single fiber has a length of about 0.5 to 1.0 μm, some even up to 1 cm, and a diameter of about 20 to 30 μm [12]. Pal has been widely used in the fields of coating materials [13], cement [14], asphalt and other building materials due to its good rheology, adsorbability and lower cost [15,16,17].

A number of studies show that the presence of nano-organic Pal can effectively improve the aging resistance of asphalt and compatibility between polymer and asphalt. Zhang et al. [10] synthesized organic-Pal under microwave irradiation and applied it to Styrene butadiene rubber (SBR)-modified asphalt. They found that organic-Pal improved the compatibility and storage stability of SBR-modified asphalt. Then, they studied the rheological and morphological properties of SBR-modified asphalt with organic-Pal, and found that organic-Pal has a positive effect on improving the viscoelasticity and anti-rutting properties of SBR-modified asphalt [18]. Sun et al. [15] applied Pal to epoxy asphalt, and found that it has a good effect on tensile and adhesive properties. Jin et al. [19] applied organic-Pal to asphalt, and found that the aging resistance of asphalt was greatly improved. At present, the effect of organic-Pal on styrene-butadiene-styrene (SBS)-modified asphalt is rarely studied. In order to further understand its effect and improve the performance of SBS-modified asphalt, this study used the star-shaped SBS modifier YH-801 and the nano-organic palygorskite (A-Pal) to prepare compounded SBS-modified asphalt. The light part of the asphalt can be adsorbed by the Pal with strong adsorption, such that the colloidal structure of the asphalt can be changed and the temperature stability of the modified asphalt can be improved [20].

## 2. Materials Preparation and Test Method

### 2.1. Materials

The 70# asphalt (AH-70) was produced by Maoming Petrochemical Co., Ltd. (Guangzhou, China) with the basic performance test results shown in Table 1. The palygorskite originated from Jiangsu, China. The basic performance parameters are shown in Table 2. The star-shaped styrene-butadiene-styrene block copolymer YH-801 (SBS4303) was produced by Yueyang Baling Petrochemical (Hunan, China) with a block ratio of 30/70.

### 2.2. Preparation of A-Pal-Compounded SBS-Modified Asphalt

Based on our previous research [19,21], Pal was treated with 1 mol/L HCl solution at 60 °C for 1 h to remove some large particles and cationics outside the raw material, then washed to neutral and dried. The treated Pal and γ-aminopropyltriethoxysilane (APTES) were dispersed in xylene solution, and the condensation reflux method was used for magnetic stirring for 10 h, then washed several times with the filtrate, dried and crushed to obtain A-Pal to enhance compatibility with the asphalt matrix. The amounts of 0 wt%, 1 wt%, 3 wt% and 5 wt% A-Pal, which composited 5 wt% of the SBS-modified asphalt, were prepared by the melt blending method (named as AH-70+5Y, AH-70+5Y+1A, AH-70+5Y+3A, and AH-70+5Y+5A, respectively).

### 2.3. Characterization

A fluorescence microscope (FM) was used to describe the phase morphology of modified asphalt with short-wave blue-purple light (λ = 420 nm) excitation (DM3000, Leica). The phase morphology of the fluorescent component in the asphalt was observed by optical microscopy to further study the correlation between microstructure and macroscopic properties [22].

### 2.4. High Temperature Rheological Evaluation

High temperature performance of asphalt refers to the ability of asphalt to resist permanent deformation under load, which was evaluated by a dynamic shear rheometer (DSR, MCR 301, Anton Paar, Austria) for temperature scanning and frequency scanning tests. The temperature scanning test was carried out in accordance with the AASHTO T315-05 [23] to study the effect of temperature change on the complex shear modulus G* and phase angle δ of A-Pal-compounded SBS-modified asphalt, with a heating rate of 2 °C/min and a temperature of 40~90 °C. Most asphalt under the working temperature of pavement belongs to the pseudo-plastic non-Newtonian fluid, and the viscosity of asphalt decreases with increasing shear rate. When the shear rate was extremely high or very small, the viscosity of the pseudo-plastic non-Newtonian fluid approached a constant, and the region where the viscosity of asphalt did not change with the shear rate was called the first Newtonian flow region and the second Newtonian flow region. The viscosity of the pseudo-plastic non-Newtonian fluid was in the first region and reached a maximum when it was constant, which is called zero shear viscosity (ZSV) [24]. The viscosity of the pseudo-plastic non-Newtonian fluid was in the second region and reached a minimum when it was constant, which is called the interfacial shear viscosity (ISV). The test results were fitted by Carreau model and calculation of ZSV [25]. The test at a temperature of 60 °C according to the AASHTO T315-05, 25 mm of oscillating plate and a film thickness of 1 mm was used for the frequency scanning test in the range of 0.01–100 Hz, and the curve was scanned by exponential growth.

### 2.5. Water Stability Evaluation

The contact angles of the A-Pal-compounded SBS-modified asphalt samples were measured by the contact angle measuring instrument (DSA100, Kruss, Germany). The sessile drop method was carried out with pure water, formamide and ethylene glycol. The surface free energy was calculated by the Owens–Wendt–Rabel–Kaelble (OWRK) method [26], and the relationship between the three was expressed by the OWRK method as follows.

(1) $$\gamma_{sl}=\gamma_{l}+\gamma_{s}-2{\left(\gamma_{l}^{d}\gamma_{s}^{d}\right)}^{1/2}-2{\left(\gamma_{l}^{p}\gamma_{s}^{p}\right)}^{1/2}$$

where $\gamma_{sl}$ is the surface free energy of the solid–liquid phase, $\gamma_{l}$ is the surface free energy of the liquid, $\gamma_{s}$ is the surface free energy of the solid, $\gamma_{l}^{d}$ is the dispersion component of the liquid, $\gamma_{s}^{d}$ is the dispersion component of the solid, $\gamma_{l}^{p}$ is the polar component of the liquid and $\gamma_{s}^{p}$ is the polar component of the solid.

Based on the surface free energy data analysis of three common mineral materials, the work of adhesion (Was) for the asphalt on the surface of the mineral material was calculated as shown in Equation (3) [27,28].

(2) $$W_{as}=\gamma_{a}+\gamma_{s}-\gamma_{as}$$

Bring Equation (1) into Equation (2) to get:

(3) $$W_{as}=2{\left(\gamma_{a}^{d}\gamma_{s}^{d}\right)}^{1/2}+2{\left(\gamma_{a}^{p}\gamma_{s}^{p}\right)}^{1/2}$$

where $\gamma_{a}^{d}$ is the dispersion component of the asphalt, $\gamma_{s}^{d}$ is the dispersion component of the mineral material, $\gamma_{a}^{p}$ is the polar component of the asphalt and $\gamma_{s}^{p}$ is the polar component of the mineral material.

The change of Gibbs free energy (ΔGaws) in each stage of spalling damage can be expressed by the work of exfoliation [29] and the calculation expression as follows:

(4) $$-\Delta G_{aws}=W_{aws}=\gamma_{aw}+\gamma_{sw}-\gamma_{as}$$

Bring Equation (1) into the above Equation to get:

(5) $$W_{asw}=2(\gamma_{w}+{\left(\gamma_{a}^{d}\gamma_{s}^{d}\right)}^{\frac{1}{2}}+{\left(\gamma_{a}^{p}\gamma_{s}^{p}\right)}^{\frac{1}{2}}-{\left(\gamma_{a}^{d}\gamma_{w}^{d}\right)}^{\frac{1}{2}}-{\left(\gamma_{a}^{p}\gamma_{w}^{p}\right)}^{\frac{1}{2}}-{\left(\gamma_{s}^{d}\gamma_{w}^{d}\right)}^{1/2}-{\left(\gamma_{s}^{p}\gamma_{w}^{p}\right)}^{1/2}$$

where $\gamma_{w}$ is the surface free energy of the water, $\gamma_{w}^{d}$ is the dispersion component of the water and $\gamma_{w}^{p}$ is the polar component of the water.

### 2.6. Aging Performance Evaluation

The aging performance of A-Pal-compounded SBS-modified asphalt was evaluated by the short-term aging, long-term aging and fatigue factor. The mass loss rate (MLR), softening point increment index (∆S), rutting factor aging index (RAI) and zero shear viscosity aging index (ZSVAI) of asphalt samples were analyzed after aging treatment in the rolling thin film oven test (TFOT) and pressure aging vessel (PAV) to simulate the short-term and long-term aging of asphalt by AASHTO R28-09 [30]. The critical temperature (fatigue limit temperature) grade corresponding to the fatigue factor (G* × sinδ >5000 kPa) was tested from the temperature fatigue test, as an index for evaluating the fatigue resistance of asphalt.

(6) $$\mathrm{RAI}=\frac{{\frac{G^{*}}{\sin\delta }}_{\left(\mathrm{aged}\right)}-{\frac{G^{*}}{\sin\delta }}_{\left(\mathrm{fresh}\right)}}{{\frac{G^{*}}{\sin\delta }}_{\left(\mathrm{fresh}\right)}}$$

(7) $$\mathrm{ZSVAI}=\frac{{\mathrm{ZSV}}_{\left(\mathrm{aged}\right)}-{\mathrm{ZSV}}_{\left(\mathrm{fresh}\right)}}{{\mathrm{ZSV}}_{\left(\mathrm{fresh}\right)}}$$

### 2.7. Low Temperature Rheological Evaluation

Low temperature performance of asphalt refers to the ability of asphalt to resist cracking under load. The low temperature crack resistance of the modified asphalt after TFOT+PAV aging was evaluated by a bending beam rheometer (BBR), in accordance with the specification AASHTO T313-12 [31]. According to the specification, 6 °C was the test range until the asphalt’s performance did not meet the requirements. The flexural creep stiffness and m value were tested under the temperatures 0, −6, −12, −18 and −24 °C with a load of 0.980 ± 0.05 N for 240 s.

## 3. Results and Discussion

### 3.1. Morphological Characteristics

**Figure 1:**
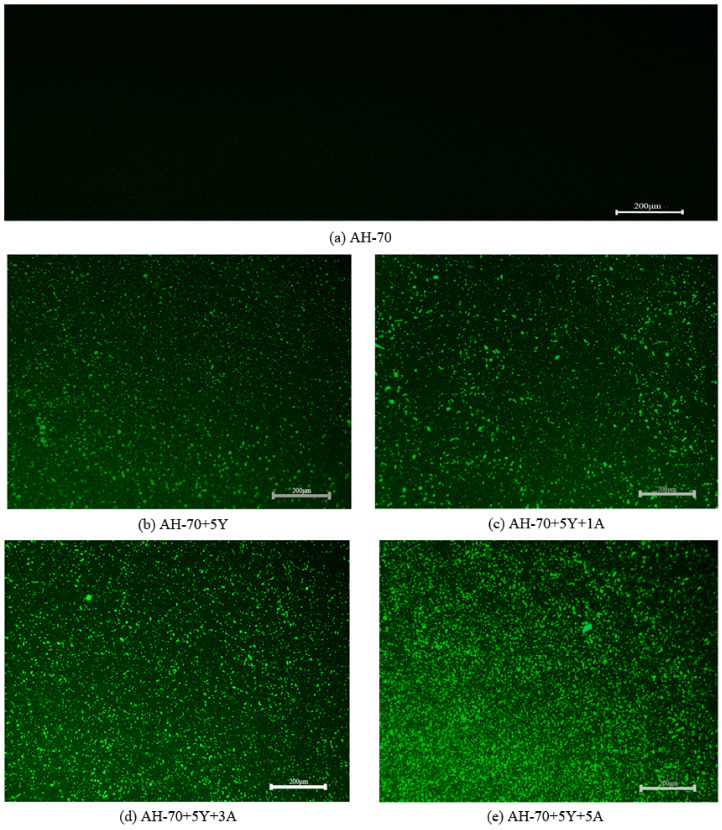
FM of A-Pal-compounded styrene-butadiene-styrene (SBS)-modified asphalt (400×): (**a**) AH-70; (**b**) AH-70+5Y; (**c**) AH-70+5Y+1A; (**d**) AH-70+5Y+3A; (**e**) AH-70+5Y+5A.

FM was carried out to observe the distribution and structure of SBS and A-Pal in the modified asphalt [32]. To enhance the discrimination between asphalt and modifiers, the asphalt part of the image is displayed as black, and the polymer part is shown as green bright spots by adjusting the brightness shown in Figure 1. Asphalt is displayed as the continuous phase, and the dispersed-phase SBS was dispersed as the form of an island in the matrix pitch [33]. Figure 1b shows a large amount of small blocky SBS crosslinks in the asphalt, which accounts for a small proportion and the scattered distribution of the asphalt without A-Pal. SBS features a low ability to absorb soft asphaltenes from asphalt, resulting in low compatibility. After adding 1 wt% A-Pal (Figure 1c), the proportion of fluorescent substances was slightly increased, and the dispersion was still unevenly distributed in the asphalt. The ability of SBS polymer to absorb soft asphaltenes after A-Pal was added had a certain increase, which leads to the volume expansion of SBS polymer and the increase in the swelling degree [22]. With the addition of A-Pal (Figure 1d,e), the proportion of fluorescent substances continues to increase, and the degree of dispersion becomes more and more uniform. After adding A-Pal, the compatibility of SBS polymer with asphalt was improved to some extent; the low temperature and fatigue performance of modified asphalt should improve [19].

### 3.2. High Temperature Performance of A-Pal-Compounded SBS-Modified Asphalt

**Figure 2:**
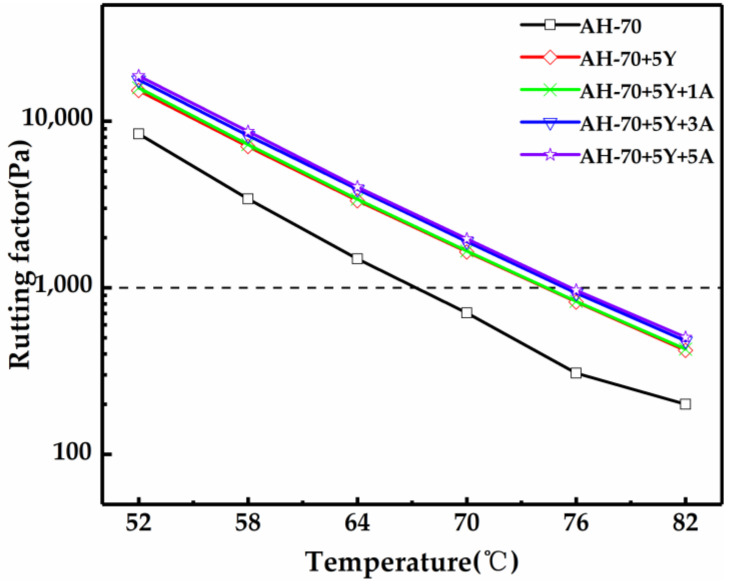
Rutting factor of A-Pal-compounded SBS-modified asphalt.

The high temperature stability is an important indicator for asphalt. The variation of the rutted factor obtained by the temperature scanning test is shown in Figure 2. It can be seen that the addition of SBS and A-Pal contributes to the improvement of the rutting factor and the rutting resistance. After SBS was added to asphalt, the rutting factor of asphalt showed a large increase and more improved resistance to rutting. The rutting factor continued to increase with the incorporation of A-Pal to further increase the rutting resistance. Compared with the previous study, it is consistent and has not changed due to the different types of SBS [18,19]. The sample with A-Pal content of 5 wt% had the highest rutting factor and the strongest anti-rutting ability, indicating that the incorporation of A-Pal can improve the temperature stability of SBS-modified asphalt. The value of the rutting factor decreases with the increasing temperature, and the rate was basically the same, indicating that all the modified asphalt samples have the same rheological properties.

The rutting factor critical temperature is the corresponding temperature factor of G*/sin δ = 1.0 kPa in the rutting factor test in the Strategic Highway Research Program (SHRP). The critical temperature of each sample is shown in Table 3. SBS could raise the critical temperature by 7.2 °C, compared with AH-70. After adding A-Pal, the rutting factor critical temperature continuously increased, and the maximum temperature increased to 75.7 °C, which was 20% higher than the matrix asphalt.

The ZSV of the modified asphalt increases with the increasing of A-Pal content, which was similar to the test result of the rutting factor (Table 4). The ZSV of the asphalt matrix increased 296% by the addition of SBS. After adding 1 wt% A-Pal, the ZSV of the modified asphalt increased to 949.4 Pa·s, which was higher than that of the modified asphalt with only SBS. With the increasing A-Pal content, the value of ZSV continues to increase, and the ZSV value of the 5 wt% compounded SBS-modified asphalt increases to 1291.8 Pa·s, which was 423% higher than that of the asphalt matrix. It showed that the compounding method was effective for improving the high-temperature stability of the asphalt binder.

### 3.3. Water Stability of A-Pal-Compounded SBS-Modified Asphalt

The contact angle of pure water, formamide and ethylene glycol between asphalt fluctuates in different degrees (Table 5). The surface free energy and its components in the compounded modified asphalt sample were calculated by the OWRK method (Table 6). Compared with the results of SBS-modified asphalt, the surface free energy of A-Pal-compounded SBS-modified asphalt together with the polar component increased. The dispersion component first rises and then falls. The work of adhesion of A-Pal-compounded SBS-modified asphalt reaches the maximum with the A-Pal amount of 1 wt%, which was 15.6% higher than that of asphalt just modified with SBS. A-Pal features a fibrous structure, which can irregularly distribute in the modified asphalt, prevent the occurrence of asphalt cracks and increase the water stability. As the A-Pal content increases, the fiber nano-material will appear as uneven agglomerations with lower dispersion [34].

**Figure 3:**
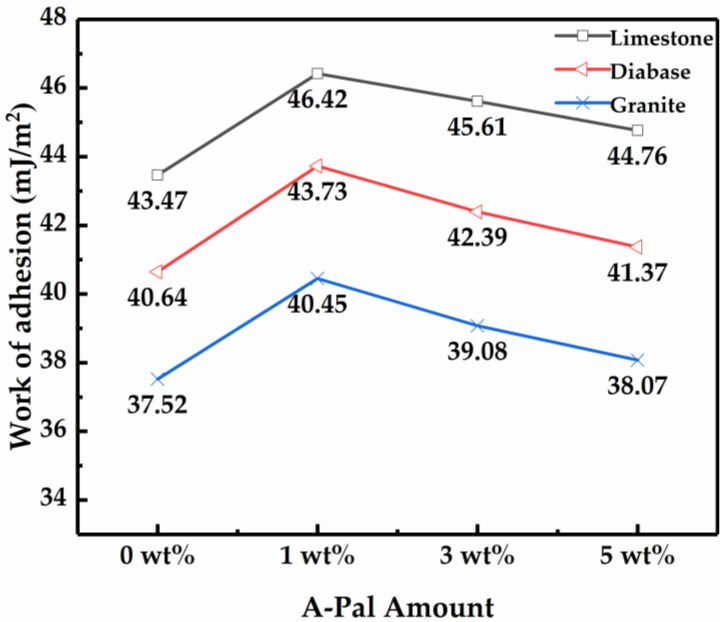
Adhesion work of modified asphalt with different amounts of A-Pal.

The adhesion of asphalt with minerals can be expressed by the work of adhesion, which can also reflect the resistance to water damage. The work of adhesion was calculated with the contact angle and surface free energy by Equation (3), shown in Figure 3. When SBS-modified asphalt was blended with 1 wt% A-Pal, the work of adhesion of the modified asphalt and the tested three mineral materials were obviously improved. Among the three minerals, the alkaline limestone has the best adhesion to the compound-modified asphalt, but the addition of A-Pal provides the greatest improvement to the adhesion with the acid aggregate granite. The addition of A-Pal enhances the interaction between asphalt and aggregate and improves the adhesion effect, which is similar to other studies [10]. With further increasing the amount of A-Pal, the work of adhesion of A-Pal-compounded SBS-modified asphalt and the three mineral materials showed different degrees of decline. The addition of A-Pal mainly affects the dispersion component in the free energy of the SBS-modified asphalt, and the effect of the polar component was not obvious [35]. As a result, the influence of modified asphalt on limestone with strong polarity is weak. With an increasing amount of A-Pal, the uneven agglomeration phenomenon affects the adhesion to modified asphalt. With the content increase, the adhesion work continues to decrease, and the adhesion performance declines.

**Figure 4:**
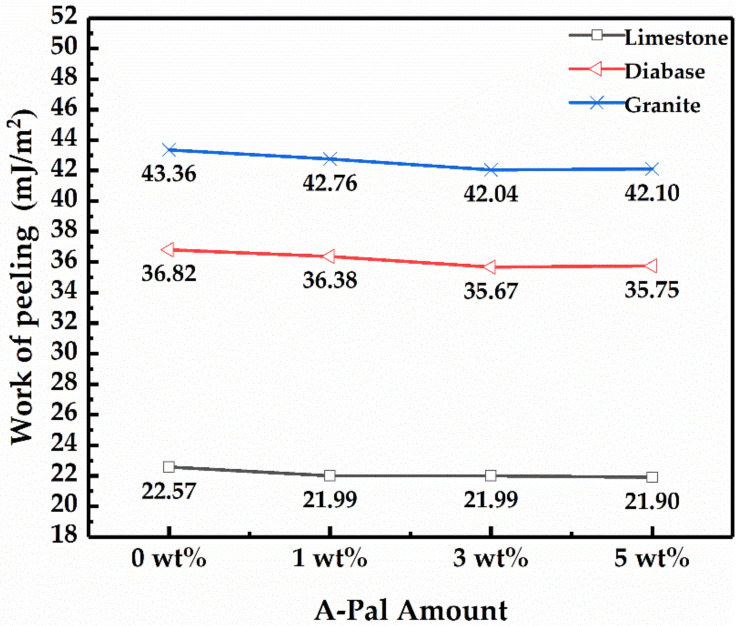
Peeling work of modified asphalt with different amounts of A-Pal.

The work of peeling (W_asw_) indicates the rate at which the asphalt spontaneously spalls from the surface of the mineral. The W_asw_ was the opposite of the Gibbs free energy change. The larger the W_asw_ is, the faster the asphalt peels off from the surface of the mineral material, and the faster water damage occurs. The W_asw_ was calculated by Equation (5), and the results are shown in Figure 4. The alkaline aggregate limestone has better anti-flaking ability and stronger water damage resistance within the test range. It has been mentioned in many research studies that alkaline aggregate has a good bonding effect with asphalt, and an obvious anti-spalling effect [18,20,27]. For the different amounts of A-Pal, the effect of A-Pal-compounded SBS-modified asphalt and mineral materials on the exfoliation work was not obvious. The peeling work was basically the same with each dosage.

### 3.4. Aging Resistance of A-Pal-Compounded SBS-Modified Asphalt

#### 3.4.1. Short-Term Aging Analysis

**Figure 5:**
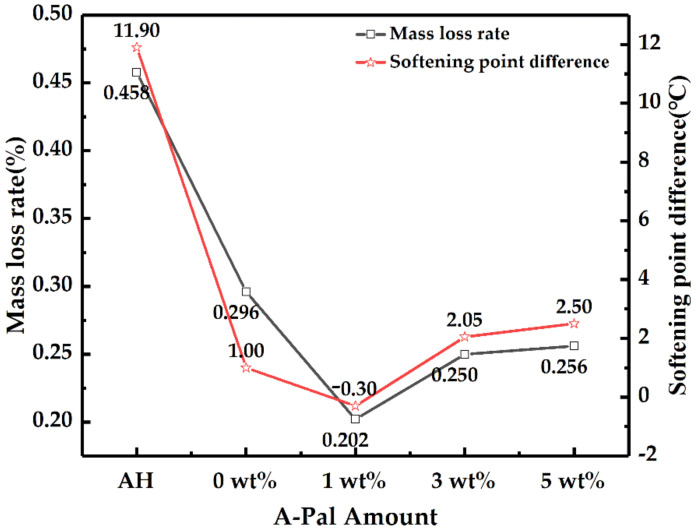
Mass loss rate and softening point of modified asphalt after thin film oven test (TFOT) aging.

The asphalt samples after the TFOT were analyzed by MLR and ∆S and are shown in Figure 5. The MLR of the modified asphalt with A-Pal content of 5 wt% was 0.202%, which was 55.9% lower than that of the SBS-modified asphalt. The adsorption and shielding of A-Pal on light components of asphalt reduced the thermal volatilization of the base asphalt and had an obvious effect on improving short-term aging, which has a similar anti-aging mechanism to clay minerals in asphalt [32,35]. With the increasing content of A-Pal, the ∆S appears to first decrease and then increase. The ∆S with A-Pal content of 1 wt% showed the minimum value of −0.3 °C, which may be due to the addition of A-Pal resulting in SBS decomposition during the short-term aging process.

**Figure 6:**
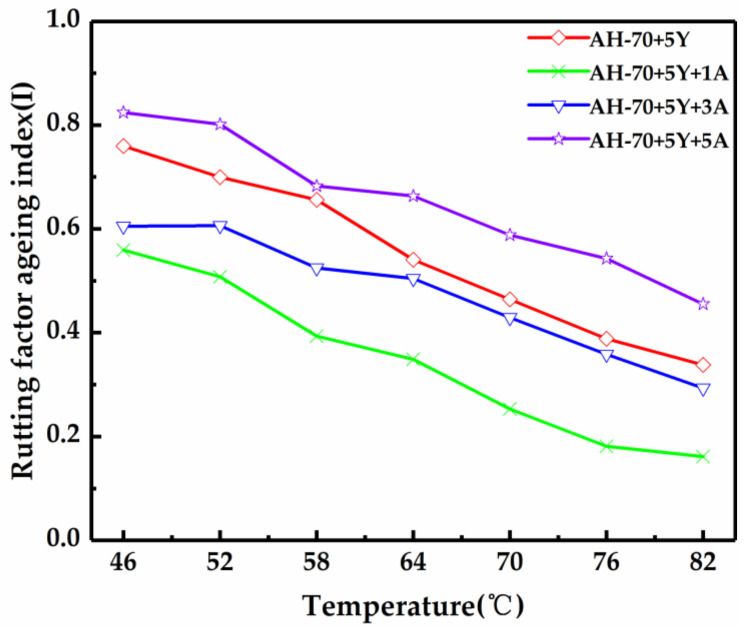
Rutting factor aging index of modified asphalt after TFOT aging.

The rheological index RAI and ZSVAI were used to evaluate the distortion of the asphalt (Figure 6 and Table 7). Due to the particularity of A-Pal-compounded SBS-modified asphalt, the polymer decomposed and the viscosity decreased during the aging progress. Meanwhile, the colloidal structure of the asphalt itself was also easily affected by aging, ranging from sol to gel, and the rheological properties of asphalt will also been changed [36]. The two indicators showed the same trend as the increasing content of A-Pal, and the sample with 1 wt% of A-Pal shows the better anti-aging property.

#### 3.4.2. Long-Term Aging Analysis

**Figure 7:**
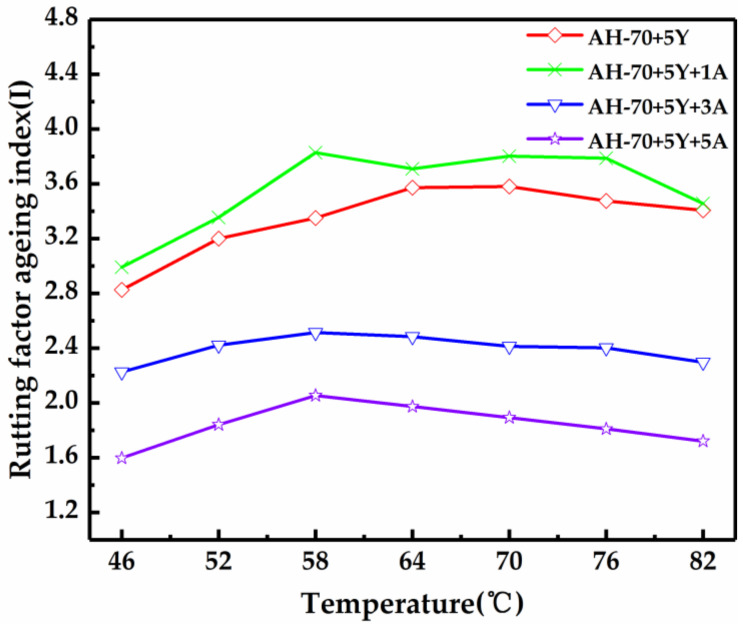
Rutting factor aging index of modified asphalt after pressure aging vessel (PAV) aging.

The long-term aging in a PAV at 100 °C as specified by AASHTO R28-09 was carried out to understand the thermal decomposition of SBS at high temperature [30]. The calculation results of RAI and ZSVAI are shown in Figure 7 and Table 8, respectively, and the two indicators showed the same trend, but contrary to the short-term aging results. After long-term aging, the RAI values of modified asphalt with 3 wt% and 5 wt% A-Pal compounded at the same temperatures were significantly lower than that of the SBS-modified asphalt, and asphalt with 5 wt% A-Pal featured the lowest RAI, indicating the best anti-aging property. The surface of SBS in modified asphalt was coated with the mineral modifier of A-Pal as a barrier, which can not only reduce the thermal aging of the asphalt matrix but can also reduce the aging decomposition of SBS. In previous studies, when mineral modifiers were applied to asphalt, the specific geometric constraints delayed the aging performance of asphalt, which indicates that A-Pal was beneficial to asphalt as a modifier [19].

#### 3.4.3. Anti-Fatigue Performance Analysis

The fatigue test results of the A-Pal-compounded SBS-modified asphalt are shown in Table 9. Asphalt samples with a lower fatigue factor critical temperature feature the better fatigue resistance. A-Pal can affect the fatigue factor to a certain extent. The 3 wt% of A-Pal-compounded SBS modified asphalt features the lower fatigue factor and better fatigue resistance. The addition of A-Pal could prevent the nucleation growth of SBS, and promote the asphalt mixture system uniformity, resulting in the enhanced fatigue resistance [35].

### 3.5. Low-Temperature Stability of A-Pal-Compounded SBS-Modified Asphalt

**Figure 8:**
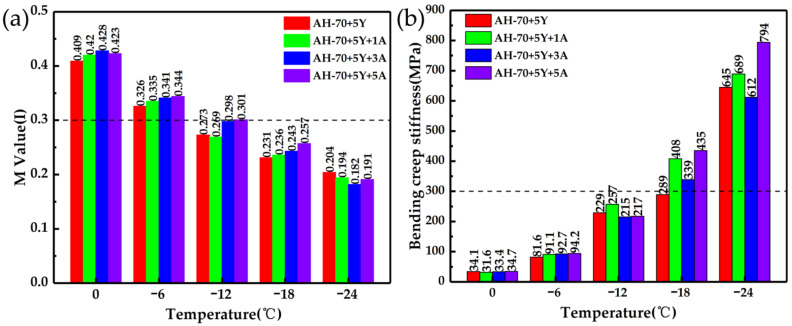
(**a**) M values and (**b**) bending creep stiffness of modified asphalt under different temperatures.

After TFOT+PAV aging, A-Pal-compounded SBS-modified asphalt was used for low-temperature crack resistance testing. The BBR test was carried out at 0, −6, −12, −18 and −24 °C for each amount of A-Pal-compounded SBS-modified asphalt with the creep rate M value shown in Figure 8. The higher the creep rate M value in the asphalt material, the smaller the tensile stress in the material, and the better the low-temperature crack resistance in the SHRP. The A-Pal-compounded SBS-modified asphalt features a higher M value than the SBS-modified asphalt at the test temperatures of 0, −6, −12 and −18 °C, indicating better flexibility and crack resistance. When the M value was greater than 0.3, the creep rate M value increased as the A-Pal content increased. Asphalt material presents brittle and hard, and the crack resistance will become worse when the bending creep stiffness of the material is too large (less than 300 MPa at 60 s, Figure 8b) [37]. Because of the fibrous one-dimensional nano-mineral, A-Pal provides a certain reinforcement effect in the asphalt, the modified asphalt becomes more viscous and the creep stiffness of A-Pal-compounded SBS-modified asphalt was higher in a certain range. The creep stiffness of each amount of A-Pal-compounded SBS-modified asphalt was not changed obviously, basically remaining at the same level within the test temperature of 0, −6 and −12 °C, and still meeting the specified stiffness requirements. The addition of A-Pal provided a certain improvement in the low-temperature performance of the star-shaped SBS modified asphalt.

## 4. Conclusions

This paper mainly studies the properties and mechanism of nano-organic palygorskite-compounded star-shaped SBS-modified asphalt. The performance of the asphalt matrix is mainly improved by SBS and, secondarily, supplementation with A-Pal. A-Pal helps to enhance the swelling degree of SBS polymer and improve the uniformity of polymer in asphalt, and the compatibility of SBS polymer with the asphalt was improved to some extent. The critical temperature of the rutting factor of the 5 wt% A-Pal-compounded SBS-modified asphalt is 20.8% higher than that of SBS-modified asphalt, which has a positive effect on improving its high-temperature stability and anti-rutting properties. Adding an appropriate amount of A-Pal to SBS-modified asphalt can increase the adhesion of modified asphalt to aggregates and the water stability of the asphalt mixture. In addition, A-Pal can reduce both the thermal aging of the asphalt matrix and the aging decomposition of the star-shaped SBS, and its aging resistance increases with the increase of the content. The comprehensive performance of 1 wt% A-Pal-compounded SBS-modified asphalt has excellent water stability and short-term aging performance, and has a slight improvement on high-temperature stability and low-temperature performance.

## Figures and Tables

**Table 1:** Test results of the 70# asphalt (AH-70) asphalt matrix.

Test Indicator	Technical Requirements	Measured Value	Test Methods
Penetration (25 °C, 100 g, 5 s) (0.1 mm)	60~80	68	ASTM D5
Penetration index PI	−1.5~+1.0	−0.77	ASTM D5
Ductility (5 cm/min, 5 °C) (cm)	≥15	19.1	ASTM D113
Softening point (Ring ball) (°C)	≥46	49	ASTM D36
Wax content (%)	≤2.2	2	ASTM D721
Density (15 °C) (g/cm^3^)	-	1.033	ASTM D70
Dynamic viscosity (60 °C), Pa·s	≥180	217	ASTM D2171

Note: ASTM = American Society for Testing and Materials.

**Table 2:** Basic performance parameters of palygorskite (Pal).

Purity	Appearance	Fineness	Viscosity	Sieve Margin	Bulk Density	pH	Moisture	Proportion
99.99%	Grey powder	325 mesh	4000 Pa·s	0.01%	0.14 g/cm^3^	7.0–9.5	<15%	0.3

**Table 3:** Rutting factor critical temperature (°C).

Sample	AH-70	AH-70+5Y	AH-70+5Y+1A	AH-70+5Y+3A	AH-70+5Y+5A
Critical temperature	67	74.2	74.4	75.3	75.7

**Table 4:** Frequency scan test results (60 °C).

Sample	ZSV (Pa·s)	ISV (Pa·s)	R^2^
AH-70	301.55	2.65 × 10^−5^	0.995
AH-70+5Y	893.24	4.65 × 10^−5^	0.997
AH-70+5Y+1A	949.4	4.68 × 10^−5^	0.996
AH-70+5Y+3A	1108.2	5.08 × 10^−5^	0.999
AH-70+5Y+5A	1291.8	5.80 × 10^−5^	0.998

**Table 5:** Contact angle test results (°).

Sample	Pure Water	Formamide	Ethylene Glycol
AH-70+5Y	106.18	92.36	100.44
AH-70+5Y+1A	103.83	92.57	96.83
AH-70+5Y+3A	104.11	94.08	98.40
AH-70+5Y+5A	104.03	93.10	97.62

**Table 6:** A-Pal-compounded SBS-modified asphalt surface energy (mJ/m^2^).

Sample	Surface Free Energy	Dispersion Component	Polar Component	Work of Adhesion
AH-70+5Y	11.56	8.5	3.06	23.12
AH-70+5Y+1A	13.37	10.14	3.23	26.74
AH-70+5Y+3A	12.03	9.4	3.63	24.06
AH-70+5Y+5A	12.60	9.03	3.57	25.2

**Table 7:** Zero shear viscosity (ZSV) aging index of modified asphalt after TFOT aging.

Sample	AH-70+5Y	AH-70+5Y+1A	AH-70+5Y+3A	AH-70+5Y+5A
ZSV of original sample	893.24	949.4	1108.2	1291.8
ZSV after TFOT	1220.8	1225.6	1636.3	1854.8
ZSVAI	0.367	0.291	0.476	0.436

**Table 8:** ZSV aging index of modified asphalt after PAV aging.

Sample	AH-70+5Y	AH-70+5Y+1A	AH-70+5Y+3A	AH-70+5Y+5A
ZSV after TFOT	1220.8	1225.6	1636.3	1854.8
ZSV after PAV	9062.3	10100	7252.5	6887.7
ZSVAI	6.423	7.241	3.432	2.713

**Table 9:** Fatigue factor critical temperature (°C).

Sample	AH-70+5Y	AH-70+5Y+1A	AH-70+5Y+3A	AH-70+5Y+5A
Critical temperature	21.6	22.3	20.9	22.0

## Data Availability

The raw data can be extracted from the provided graphs in SI or is available from the authors upon request.
